# A null mutation in *ANGPTL8* does not associate with either plasma glucose or type 2 diabetes in humans

**DOI:** 10.1186/s12902-016-0088-8

**Published:** 2016-01-28

**Authors:** Katharine R. Clapham, Audrey Y. Chu, Jennifer Wessel, Pradeep Natarajan, Jason Flannick, Manuel A. Rivas, Samantha Sartori, Roxana Mehran, Usman Baber, Valentin Fuster, Robert A. Scott, Daniel J. Rader, Michael Boehnke, Mark I. McCarthy, David M. Altshuler, Sekar Kathiresan, Gina M. Peloso

**Affiliations:** Department of Medicine, Massachusetts General Hospital, Harvard Medical School, Boston, MA 02114 USA; Division of Preventive Medicine, Brigham and Women’s Hospital, Boston, MA 02215 USA; National Heart, Lung, and Blood Institute (NHLBI) Framingham Heart Study, Framingham, MA 01702 USA; Department of Epidemiology, Fairbanks School of Public Health, Indianapolis, IN 46202 USA; Department of Medicine, Indiana University School of Medicine, Indianapolis, IN 46202 USA; Center for Human Genetic Research, Massachusetts General Hospital, Boston, MA 02114 USA; Cardiovascular Research Center, Massachusetts General Hospital, Boston, MA 02114 USA; Program in Medical and Population Genetics, Broad Institute, Cambridge, MA 02142 USA; Department of Molecular Biology, Massachusetts General Hospital, Boston, MA 02114 USA; Cardiovascular Institute, Mount Sinai Medical Center, Icahn School of Medicine, Mount Sinai, New York, NY USA; MRC Epidemiology Unit, University of Cambridge School of Clinical Medicine, Institute of Metabolic Science, Cambridge Biomedical Campus, Cambridge, CB2 0SL UK; Perelman School of Medicine, University of Pennsylvania, Philadelphia, PA 19104 USA; Center for Statistical Genetics, Department of Biostatistics, University of Michigan, Ann Arbor, MI USA; Wellcome Trust Centre for Human Genetics, University of Oxford, Oxford, UK; Oxford Centre for Diabetes, Endocrinology, and Metabolism, Churchill Hospital, University of Oxford, Oxford, UK; Oxford NIHR Biomedical Research Centre, Churchill Hospital, Oxford, UK; Department of Genetics, Harvard Medical School, Boston, MA 02115 USA; 801 Massachusetts Ave, Crosstown Center, Third Floor, Boston, MA 02118 USA

**Keywords:** Betatrophin, Angiopoietin-like 8, rs145464906

## Abstract

**Background:**

Experiments in mice initially suggested a role for the protein angiopoietin-like 8 (ANGPTL8) in glucose homeostasis. However, subsequent experiments in model systems have challenged this proposed role. We sought to better understand the importance of *ANGPTL8* in human glucose homeostasis by examining the association of a null mutation in ANGPTL8 with fasting glucose levels and risk for type 2 diabetes.

**Methods:**

A naturally-occurring null mutation in human *ANGPTL8* (rs145464906; c.361C > T; p.Q121X) is carried by ~1 in 1000 individuals of European ancestry and is associated with higher levels of plasma high-density lipoprotein cholesterol, suggesting that this mutation has functional significance. We examined the association of p.Q121X with fasting glucose levels and risk for type 2 diabetes in up to 95,558 individuals (14,824 type 2 diabetics and 80,734 controls).

**Results:**

We found no significant association of p.Q121X with either fasting glucose or type 2 diabetes (*p*-value = 0.90 and 0.65, respectively). Given our sample sizes, we had >98 % power to detect at least a 0.23 mmol/L effect on plasma glucose and >95 % power to detect a 70 % increase in risk for type 2 diabetes.

**Conclusion:**

Disruption of *ANGPTL8* function in humans does not seem to have a large effect on measures of glucose tolerance.

**Electronic supplementary material:**

The online version of this article (doi:10.1186/s12902-016-0088-8) contains supplementary material, which is available to authorized users.

## Background

In type 1 and to a lesser extent in type 2 diabetes, the insulin-producing beta cells of the pancreas undergo destruction and exhibit decreased function. Recovered insulin production by abrogating this process has important therapeutic implications for individuals suffering from diabetes mellitus. A study in mice demonstrated that pancreatic beta cell proliferation is stimulated by a 198-amino acid protein named betatrophin (also known as Lipasin and ANGPTL8), leading to improved glucose tolerance [1]. Injection of betatrophin-expressing plasmids into mice resulted in increased beta cell replication and ultimately increased beta cell number and pancreatic beta cell mass. However, subsequent murine experiments of betatrophin have largely been neutral with respect to glucose alterations and overexpression of ANGPTL8 showed no abnormalities in glucose homeostasis [2, 3]. In addition, *Angptl8*^*-/-*^ mice did not demonstrate impaired glucose tolerance [2, 4]. Although its role in glucose homeostasis is still being elucidated, ANGPTL8 is known to have a role in the regulation of lipid metabolism, possibly through activation of a related protein, angiopoietin-like 3 [5]. Several experiments have shown that *Angptl8* null mice have lower triglyceride levels and conversely, that overexpression of ANGPTL8 increases triglyceride levels [2, 4, 5]. Taken together, findings in model systems suggest that whereas a role for ANGPTL8 in beta cell proliferation is uncertain, ANGPTL8 is consistently linked to triglyceride concentrations.

Null mutations can be used to inform on the effect of a gene in humans. We have previously reported a naturally-occurring null mutation in human *ANGPTL8* (rs145464906; c.361C > T; p.Q121X). *ANGPTL8* p.Q121X is estimated to be carried by 1 in 1000 individuals of European ancestry and 1 in 10,000 African-Americans [6]. Plasma high-density lipoprotein cholesterol is 10 mg/dl higher (*p*-value = 5 x 10^-11^) and triglyceride concentration is 15 % lower (*p*-value = 0.003) in carriers of *ANGPTL8* p.Q121X [6].

The lipid phenotype demonstrated by carriers of *ANGPTL8* p.Q121X suggests that this naturally occurring mutation has functional significance and allows us to test the hypothesis that carrying *ANGPTL8* p.Q121X also perturbs glucose homeostasis in humans. Previously, we have shown that *ANGPTL8* p.Q121X was not associated with fasting glucose in two studies (n < 15,000) [6]. Here, we aimed to determine the association of *ANGPTL8* p.Q121X with two measures of glucose homeostasis – fasting glucose level and type 2 diabetes status in up to 95,558 individuals (14,824 type 2 diabetics and 80,734 controls).

## Methods

### In silico analysis

*In silico* analysis was performed to predict the effect of *ANGPTL8* p.Q121X (rs145464906) mutation on transcription. We predicted whether the *ANGPTL8* p.Q121X mutation resulted in nonsense mediated decay using RNA sequencing transcript isoform data publically available from the Genotype-Tissue Expression (GTEx) project (http://www.gtexportal.org/) and a predictive model implemented in MAMBA (http://www.well.ox.ac.uk/~rivas/mamba) [7, 8].

### Study participants

Analysis was limited to subjects of self-reported European ancestry. Studies contributing summary results included the BioImage Study [9], the Cohorts for Heart and Aging Research in Genomic Epidemiology (CHARGE) Consortium Diabetes Working Group [10], and studies from the T2D-GENES consortium with > 5 minor alleles for *ANGPTL8* p.Q121X (KORA and UK) [11]. The BioImage Study and the KORA and UK studies from the T2D-GENES consortium have been approved by the MIT IRB (#1010004095, #0912003615 #1107004579, respectively). The CHARGE Diabetes Working Group consists of 27 European Ancestry studies and all participating studies were approved by local institutional review committees. All subjects have provided written informed consent.

### Genotyping and quality control

All study participants were genotyped on the HumanExome BeadChip v.1.0 or v1.1 (Illumina) for *ANGPTL8* p.Q121X and called using joint calling [12], GenomeStudio or zCall [13]. Quality control involved checking concordance to GWAS data and excluding those individuals missing >5 % genotypes, population clustering outliers, individuals with high inbreeding coefficients or heterozygote rates, individuals with gender mismatches, one individual from duplicate pairs, and individuals with an unexpectedly high proportion of identity-by-descent sharing, with consideration for family studies, based on high-quality variants. All contributing studies used an additive coding of variants to the minor allele.

### Phenotypes

Association analyses were performed for fasting glucose and type 2 diabetes individually by cohort and summary statistics were shared. In the BioImage Study, type 2 diabetes was defined as individuals taking a medication for diabetes, having a fasting glucose > 126 mg/dl or having been told that he/she had diabetes. Type 2 diabetes was defined in the CHARGE Consortium Diabetes Working Group according to Wessel et al [10], and in the T2D-GENES Consortium according to Voight et al [14]. Individuals with a diagnosis of diabetes were excluded from the fasting glucose and insulin analyses to avoid the variable effects of diabetes medications on the quantitative traits.

### Statistical analyses

Two primary analyses were performed separately by study in available participants: (1). linear regression of fasting glucose levels with an additive coding of *ANGPTL8* p.Q121X adjusting for age, sex, and principal components of ancestry; and (2). logistic regression of type 2 diabetes with an additive coding of *ANGPTL8* p.Q121X adjusting for age, sex, and principal components of ancestry. Counts of the number of *ANGPTL8* p.Q121X mutation carriers were obtained in type 2 diabetes cases and controls. Additionally, in 47,388 we analyzed the association of *ANGPTL8* p.Q121X with fasting insulin levels adjusted for BMI, age, sex, and principal components of ancestry.

For the outcomes of fasting glucose and fasting insulin levels, statistical evidence from each study was combined using fixed-effects inverse variance meta-analysis. Heterogeneity of effects between studies was ruled out (p > 0.05).

For the outcome of type 2 diabetes, we performed meta-analyses by using Cochran-Mantel-Haenszel statistics for stratified 2x2 tables. The Cochran-Mantel-Hanszel method combines score statistics rather than Wald statistics. As an alternate approach, we performed a fixed-effects inverse variance meta-analysis on the betas and standard errors from the individual study results. Using a fixed-effects inverse variance meta-analysis did not change the results.

All analyses were performed using the software program R version 2.15. We estimated statistical power to detect association of *ANGPTL8* p.Q121X with fasting glucose and type 2 diabetes using the Genetic Power Calculator (http://pngu.mgh.harvard.edu/~purcell/gpc/) [15].

## Results

The lipid phenotype previously demonstrated by carriers of *ANGPTL8* p.Q121X suggests that this naturally occurring mutation has functional significance. In addition, using *in silico* predictions, we found that *ANGPTL8* p.Q121X results in nonsense mediated decay of the RNA transcript and partial knockdown of the resulting protein (Additional file 1: Figure S1).

All participants were of self-reported European ancestry. 69,854 individuals without type 2 diabetes were available for the analysis of fasting glucose levels. In analysis for type 2 diabetes status, 14,824 individuals with a diagnosis of type 2 diabetes were compared to 80,734 controls (Additional file 1: Table S1). 24 (0.16 %) of the type 2 diabetes cases were heterozygous carriers of the minor allele for *ANGPTL8* p.Q121X whereas 142 (0.18 %) of the controls were heterozygous carriers.

**Fig. 1:**
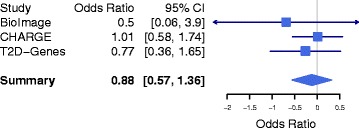
Association of *ANGPTL8* p.Q121X with type 2 diabetes in 14,824 cases and 80,734 controls. In each study, we tested the association of carrier status with type 2 diabetes. Meta-analysis was performed using the Cochran-Mantel Haensel statistics for stratified 2-by-2 tables

We combined evidence from all available studies and performed a fixed-effects meta-analysis for the association of p.Q121X with fasting glucose levels. We found no evidence for association of p.Q121X with fasting glucose levels (beta = 0.002 mmol/L; 95 % conference interval[CI] = (-0.025, 0.029); *p*-value = 0.90) (Table 1). For the outcome of type 2 diabetes, we used Cochran-Mantel-Haenszel meta-analysis to combine the counts across the studies given the low number of type 2 diabetes cases with the p.Q121X mutation. We found no evidence for association of p.Q121X with type 2 diabetes (Fig. 1, OR = 0.88, 95 % CI = 0.57 – 1.36, *p*-value = 0.65).

**Table 1:** Association of *ANGPTL8* p.Q121X with plasma glucose

Study	N	Effect size	Standard error	*p*
BioImage	4,072	0.080	0.19	0.70
CHARGE	49,838	-0.005	0.05	0.92
T2D-GENES	15,944	0.002	0.01	0.89
Meta-analysis	69,854	0.002	0.01	0.90

Additionally, we found no evidence of association for fasting insulin levels adjusted for BMI with *ANGPTL8* p.Q121X in 47,388 individuals with fasting insulin available within CHARGE (3 % change; *p* = 0.43).

We estimated statistical power to detect each of these associations given our analyzed sample sizes. Power calculations indicate that with a sample size of 69,854 we had >98 % power to detect a 0.23 mmol/L (1/2-standard deviation; 4.2 mg/dL) effect of p.Q121X on fasting glucose at an alpha level of 0.05. Given 14,824 type 2 diabetes cases and 80,734 controls, we have >95 % power to detect a genotype relative risk for carriers of > 1.7 and > 85 % power to detect a genotypic relative risk for carriers of > 1.6 at an alpha level of 0.05.

## Discussion

We find that carrying a null mutation in human *ANGPTL8* is neither associated with fasting glucose nor risk for type 2 diabetes in analyses involving up to 95,558 individuals. These results are consistent with findings from a preliminary analysis of individuals carrying *ANGPTL8* p.Q121X with fasting glucose in Peloso, et al [6], in which no significant association between *ANGPTL8* p.Q121X and fasting glucose levels was identified in <15,000 individuals, which only had 45 % power to detect a 0.23 mmol/L (1/2-standard deviation; 4.2 mg/dL) effect of p.Q121X on fasting glucose at an alpha level of 0.05. However, the larger sample size utilized in our study allowed for 98 % power and a better estimation of the impact of *ANGPTL8* p.Q121X on fasting glucose levels. This study also expands upon the phenotyping of the carriers of this allele in that we were able to examine association of *ANGPTL8* p.Q121X with a diagnosis of type 2 diabetes and with fasting insulin levels, and to ask a targeted hypothesis that may not reach an exome chip-wide significance level (*p* < 3 × 10^−7^ for fasting glucose and *p* < 4.5 × 10^−7^ for type 2 diabetes) as was explored in Wessel, et al [10].

While our results do not support the role of ANGPTL8 inhibitors for treatment of type 2 diabetes, there remains evidence that ANGPTL8 inhibition remains a viable anti-triglyceride target [2, 6]. The human genetic analysis presented here provides evidence that an ANGPTL8 inhibitor aimed at lowering plasma triglyceride levels will not have a major effect on glucose tolerance in humans.

Some limitations deserve mention. The initial therapeutic hypothesis from model systems related to gain of gene function; loss of gene function as assessed by p.Q121X may not necessarily lead to phenotypic effects in a direction opposite to gain of gene function. In addition, due to the scarcity of the allele, we were able to assess only heterozygous carriers of the allele. It is possible that there is a recessive effect on fasting glucose levels or type 2 diabetes status that would be unmasked only in individuals homozygous for the *ANGPTL8* mutation. The scarcity of the null mutation also limits our statistical power, and we are unable to rule out modest effects on glucose and type 2 diabetes.

## Conclusion

While ANGPTL8 has been shown to robustly associate with plasma triglyceride levels, our results do not support a role for ANGPTL8 in glucose homeostasis in humans.

## Additional file

Additional file 1: Supplemental Information.
**Figure S1.** *In-silico* predictions of the impact of premature termination codon p.Q121X suggests that the variant would trigger nonsensemediated decay. **Table S1.** *ANGPTL8* p.Q121X and type 2 diabetes status by study. (PDF 145 kb)
