# Supplementary material for: A null mutation in ANGPTL8 does not associate with either plasma glucose or type 2 diabetes in humans
Source: BMC Endocr Disord. 2016 Jan 28;16:7. doi: 10.1186/s12902-016-0088-8 (PMC4730725; doi:10.1186/s12902-016-0088-8)
Supplement: Additional file 1: Supplemental Information. — Figure S1. In-silico predictions of the impact of premature termination codon p.Q121X suggests that the variant would trigger nonsensemediated decay. Table S1. ANGPTL8 p.Q121X and type 2 diabetes status by study. (PDF 145 kb) [file 12902_2016_88_MOESM1_ESM.pdf]

Supplementary Figure 1. *In-silico* predictions of the impact of premature termination codon p.Q121X suggests that the variant would trigger nonsense-mediated decay. Transcript isoform ENST00000252453 is highly expressed in liver with some usage of ENST00000397785 which is a lowly expressed isoform. ENST00000252453 supports the p.Q121X annotation and results in nonsense mediated decay using RNA sequencing transcript isoform data publically available from the Genotype-Tissue Expression (GTEx) project (http://www.gtexportal.org/) and a predictive model implemented in MAMBA (http://www.well.ox.ac.uk/~rivas/mamba). Y-axis is Isoform expression value (RPKM).

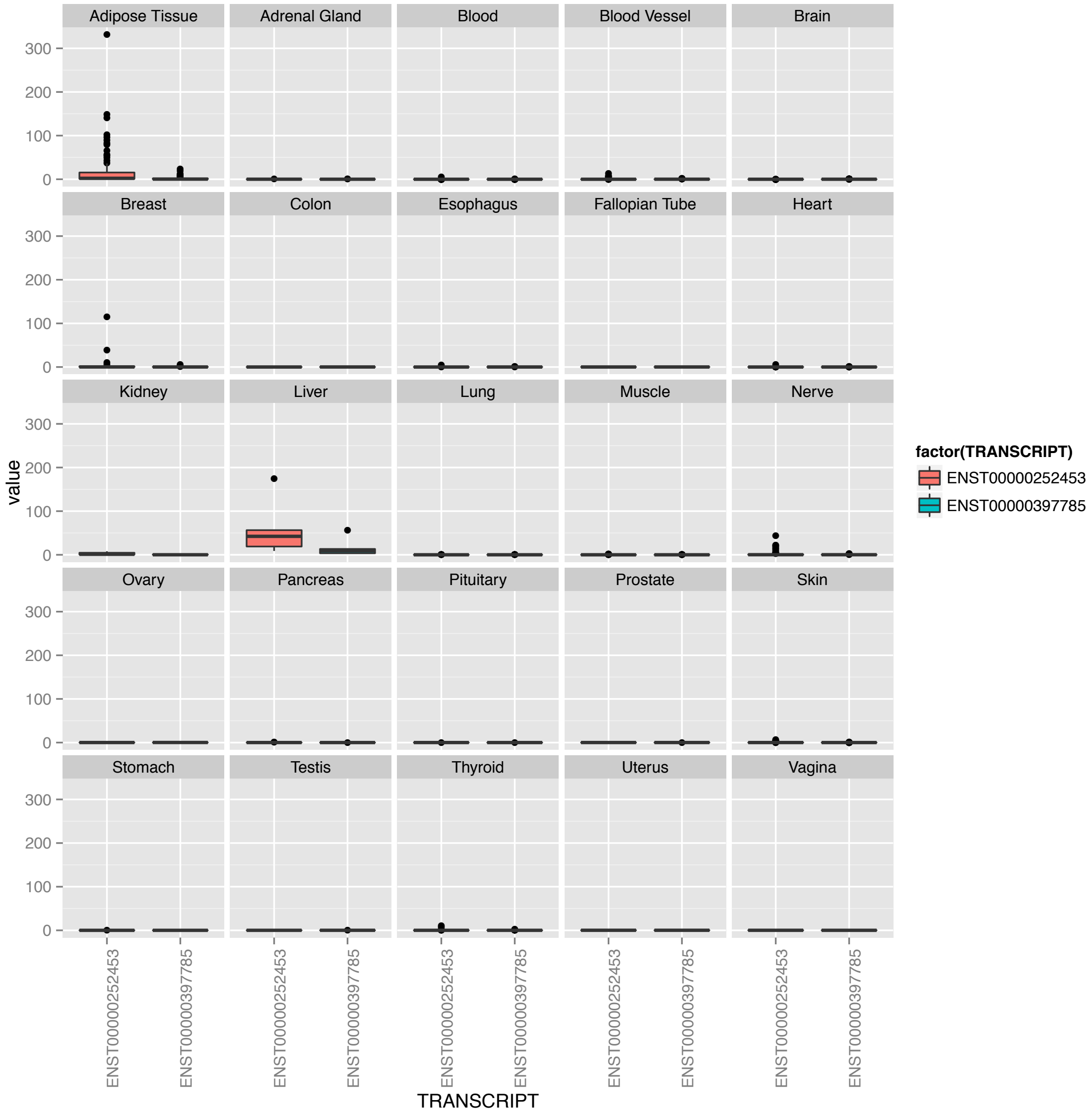

Supplementary Table 1. ***ANGPTL8*** **p.Q121X and type 2 diabetes status by study**

Study	p.Q121X carriers among cases	Percentage of cases who carry p.Q121X	p.Q121X carriers among controls	Percentage of controls who carry p.Q121X	Non-carriers among cases	Non-carriers among controls	Ref.
BioImage	1	0.12%	10	0.25%	814	4,062	[1]
CHARGE	15	0.16%	95	0.16%	9,509	60,623	[2]
T2D-GENES	8	0.18%	37	0.23%	4,477	15,907	[3]
Summary	24	0.16%	142	0.18%	14,800	80,592	

References

1. Muntendam P, McCall C, Sanz J, Falk E, Fuster V: **The BioImage Study: novel approaches to risk assessment in the primary prevention of atherosclerotic cardiovascular disease--study design and objectives**. *Am Heart J* 2010, **160**(1):49-57 e41.
2. Wessel J, Chu AY, Willems SM, Wang S, Yaghootkar H, Brody JA, Dauriz M, Hivert MF, Raghavan S, Lipovich L *et al*: **Low-frequency and rare exome chip variants associate with fasting glucose and type 2 diabetes susceptibility**. *Nature communications* 2015, **6**:5897.
3. Flannick J, Thorleifsson G, Beer NL, Jacobs SB, Grarup N, Burtt NP, Mahajan A, Fuchsberger C, Atzmon G, Benediktsson R *et al*: **Loss-of-function mutations in SLC30A8 protect against type 2 diabetes**. *Nat Genet* 2014, **46**(4):357-363.
